# Coach Turnover in Top Professional Brazilian Football Championship: A Multilevel Survival Analysis

**DOI:** 10.3389/fpsyg.2019.01246

**Published:** 2019-06-06

**Authors:** Alexandre B. Tozetto, Humberto M. Carvalho, Rodolfo S. Rosa, Felipe G. Mendes, Walan R. Silva, Juarez V. Nascimento, Michel Milistetd

**Affiliations:** ^1^Department of Physical Education, College of Health and Sport Science, Santa Catarina State University, Florianópolis, Brazil; ^2^Department of Physical Education, School of Sports, Federal University of Santa Catarina, Florianópolis, Brazil

**Keywords:** employment, work performance, soccer, proportional hazards models, bayesian methods

## Abstract

In this study, we examined the probability of coaches’ survival in the top Brazilian professional football championship considering variation across the competitive seasons between 2012 and 2017, considering a multilevel framework. We also considered whether previous coaching experience in the top Brazilian professional football championship would change the probability of coaches’ survival across the season. The data considered 4,560 games from the top professional Brazilian football league (Campeonato Brasileiro Série A) between the 2012 and 2017 seasons. At the start of each season, the coach from each team was followed, being recorded at the time the event occurred, i.e., the coach being sacked. A total survival of 120 coaches was considered between the seasons of 2012 and 2017, i.e., 20 coaches at the beginning of each season. Coaches were assigned as novice (no previous experience as head coach in the top Brazilian championship) or experienced (with at least some previous experience as head coach in the top Brazilian championship). Data were available and extracted from the official website of the Brazilian Football Confederation^1^. On average and considering un-pooled observations, the median life of a coach was about 16.5 rounds. Considering variation between 2012 and 2017 seasons, only about 26.3% (95% CI: 18.2–36.1) of the coaches ended a season without being sacked. By mid-season, at round 19, the probability of coaches’ survival was 0.42 (95% CI: 0.32–0.53). Variation between season on survival estimates per round was substantial (between-season standard deviation = 0.48, 95% credible intervals: 0.25–0.95; corresponding to an inverse logit = 0.62, 95% CI: 0.56–0.72). There was no substantial variation between novice and experienced coaches’ survival probability. The present results expose the vulnerability of the coaching context in Brazilian football, potentially highlighting an excessive emphasis on short-term results to mediate club management decisions.

## Introduction

Sports coaching is a relatively new professional field in comparison to other professions, such as medicine, law, or teaching (Mallett and Lara-Bercial, 2016). Particularly in football, in the past few decades, the vocation of sports coaching has continued to develop toward professionalization across the world.

High performance football coaches play a central role in the coach-athlete-performance relationship (Lyle, 2002; Mallett and Lara-Bercial, 2016). Coaches often face challenges and constraints that influence their daily practice and the competitive outcome. These challenges often include increased number of international competitions, the increased importance of the stakes relative to the club investment in the football team, discrepancy between available resources and competitive expectations (Mallett and Lara-Bercial, 2016). Nevertheless, high performance football coaches are evaluated for wins or losses, potentially leading to a high coach turnover (Duffy et al., 2011; Mallett and Lara-Bercial, 2016). Hence, the development of the high-performance football coach professional activity is developed within a dynamic, complex, and demanding context.

Observations in European top football leagues have shown a consistent trend of high coach turnover rate (Barros et al., 2009; Lago-Peñas, 2011; Paola and Scoppa, 2012; Bell et al., 2013; van Ours and van Tuijl, 2016), despite the negative impacts of coaches’ changes (Balduck et al., 2010; Lago-Peñas, 2011; Paola and Scoppa, 2012; van Ours and van Tuijl, 2016). It appears that coaches not meeting expectations in the first games or when results are worse than the results from the predecessor coach, have a higher likelihood of being sacked (Mallett and Lara-Bercial, 2016; van Ours and van Tuijl, 2016). Also, coaches turnover is noted to have a negative impact for the development of players and team performance after coaches’ turnover (Barros et al., 2009; Mallett and Rynne, 2015; Mallett and Lara-Bercial, 2016). On the other hand, coaches’ turnover also has potential negative impacts on the financial safety of sports clubs (Bell et al., 2013; Mallett and Lara-Bercial, 2016). For example, it has been reported that coaches’ dismissal in Australian football had a net cost of about $11 million in payouts within a 5-year period (Mallett and Lara-Bercial, 2016).

Brazil is the country with the most wins in the FIFA World Cup (Hoffmann et al., 2002) and is consistently among the highest ranking countries by the Fédération Internationale de Football Association (Hoffmann et al., 2002; FIFA.com, 2019). Football is a highly relevant socioeconomic activity in Brazil, and the activity of football coaching, in particular, is recognized specifically as a profession by law (Brazil, 1993). However, in the Brazilian context, there are limited available data addressing the conflict of interest between the high performance football coach’s survival, the team’s long-term preparation, and the need for short-term club results, influenced by industry specificities such as media and sports fans pressure.

To the best of our knowledge, research questions and designs dealing with occurrence and timing of critical events in sports and exercise research are scarce. Event history analysis (also known as survival analysis) is used to study the timing of events where the response variable is the length of time between becoming exposed to the risk of an event and event occurrence (Steele, 2011). Changes in coaches’ job status is an example of observations of each coach’s time of survival to the risk of being sacked. These data can be viewed as a type of two-level hierarchical structure with episodes of being at risk of an event nested within individuals, and individuals may themselves be nested by competitive season. When considering events such as a coach being sacked, coaches’ survival probabilities may vary substantially by competitive seasons due to potential influences on their daily practice and the competitive outcome. Multilevel modeling framework provides a flexible approach for the analysis of hierarchical (and nonhierarchical) structures, in particular in the case of clustered event history data (Goldstein, 2011). Therefore, in the present study, we examined the probability of coaches’ survival in the top Brazilian professional football championship considering variation across the competitive seasons between 2012 and 2017, considering a multilevel framework. We also considered whether variation between head coaching experience in the top Brazilian professional football championship would influence the survival probability across the seasons.

## Materials and Methods

### Data

The data in the present study are based on observations of 4,560 games from the top professional Brazilian football league (Campeonato Brasileiro Série A) between the 2012 and 2017 seasons. Hence, the period of observation comprised six seasons. The Serie A Brazilian football league is composed by 20 teams. Each plays 38 matches per season (i.e., 38 rounds), and at the end of the competition, the four teams with the lowest score are relegated and replaced by the four best teams of the second division. Considering variation between seasons in the teams entering the league, we considered 38 rounds of the season as the time unit of observation. At the start of each season, the coach from each team was followed, being recorded at the time event occurred, i.e., the coach being sacked. A total survival of 120 coaches was considered between the seasons of 2012 and 2017.

Data were extracted from the 4,560 match reports for the seasons between 2012 and 2017, available on the official website of the Brazilian Football Confederation (see text footnote 1). Considering the available information, we assigned coaches by experience as novice, i.e., with no previous experience as head coach in the top Brazilian championship, or experienced, i.e., with at least a previous experience as head coach in the top Brazilian championship. From the 120 coaches observed, 17 were assigned as novice, and the remaining 103 were assigned as experienced.

### Data Analysis

Given that the unit of time comprises the 38 rounds in each season, we assumed the possibility for right censoring. Right censoring occurs in survival analysis when some individuals never experience the target event during the period of observation (Singer and Willett, 2003). In the present study, the follow-up of the coaches that did not experience the event at the end of the season was not considered in the present analysis, whether the coaches remained in the same team competing in the Serie A, remained in the same team if the team was relegated to the lower division, or changed to another team after the end of the season.

The survival function gives the probability that a randomly selected coach survives longer than some specified time period (in the present study, each league round). Hence, the survivor function is generally given by the number of individuals who have not experienced the event by the end of each time period divided by the number of individuals in the data set (Singer and Willett, 2003). Initially, the survival function was estimated for each coach without accounting for coach turnover variation by season using an un-pooled logistic regression. Hence, each round across the Brazilian top professional football league between 2012 and 2017, without accounting for variation between seasons, had its own intercept with a simple aggregated binomial model, described following a Bayesian notation:

**Table:** 

surv*_i_* ∼ Binomial(*n*_[*i*]_, *p*_[*i*]_)	[likelihood]
logit(*p_i_*) = *α*_[*i*]_	[unique log-odds for each round *i*]
*α* ∼ Normal(0,1)	[weakly regularizing prior]

Considering the hierarchical data, where each coach observation occurs within a higher order entity (between season), we then described the survival probability across the 38 rounds within the seasons using Bayesian multilevel modeling to estimate a partial pooling model. The multilevel model was produced as follows:

**Table:** 

Surv*_i_* ∼ Binomial(*n*_[*i*]_, *p*_[*i*]_)	[likelihood]
logit(*p*_[*i*]_) = *α*_SEASON[*i*]_	[unique log-odds for each round *i* by season]
*α*_SEASON_∼ Normal(*α*,*σ*)	[varying intercepts prior]
*α* ∼ Normal(0,1)	[prior for average season]
*σ* ∼ Cauchy(0,1)	[prior for standard deviation of season]

Survival probabilities were extracted from both binomial models using the inverse logit function (Gelman and Hill, 2007; McElreath, 2015).

In the final step of the analysis, we examined the influence of previous experience as head coach on the top Brazilian professional championship. We considered the main effects for the proportion of novice coaches by experienced coaches at each round per season as population-level effect, plus the interaction with each round. We allowed for the proportion between novice and experienced coaches at each round to vary randomly by season at level 2.

We used weakly informative prior distributions for population-level, normal priors (0,1), and for group-level, Cauchy priors (0,1), effects. Posterior predictive checks were used to confirm that we did not omit relevant interactions (Gelman et al., 2013). Comparisons between un-pooled (single-level model) and partial pooled (multilevel model) models were made with the widely applicable information criteria (WAIC) (McElreath, 2015; Vehtari et al., 2016). For each model, we ran two chains for 4,000 iterations with a warm-up length of 1,000 iterations. The models were implemented with Markov Chain Monte Carlo (MCMC) simulation, *via* Hamiltonian Monte Carlo using Stan (Stan Development Team, 2015), obtained using “brms” package (Bürkner, 2017), available as a package in the R statistical language.

## Results

From the 120 coaches in the competitive top Brazilian football league between 2012 and 2017, 87 coaches were sacked during a season. On average and considering un-pooled observations, the median life of a coach was about 16.5 rounds.

Models’ codes and respective summaries, trace plots to ascertain chains’ convergence, and posterior predictive checks are presented as Supplementary Material. WAIC was substantially lower for the partial pooled model (WAIC = 847.99, standard error = 9.76) in contrast to un-pooled model (WAIC = 990.66, standard error = 21.60). Partial pooled model performed substantially better fitting the data when compared to un-pooled model.

**Figure 1:**
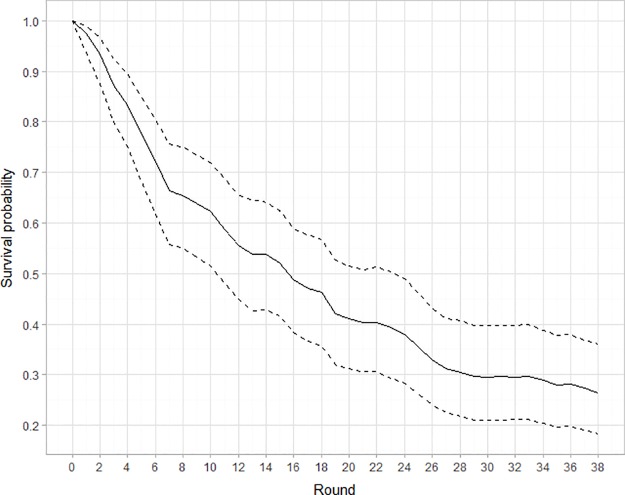
Sample survival function for round at coach dismissal among 120 coaches between 2012 and 2017.

Results from the partial pooled models, i.e., a multilevel logistic model to estimate the football coaches’ survival across the 38 rounds in the Brazilian top professional league, are presented in Table 1 and Figure 1. Considering variation between 2012 and 2017 seasons, only about 26.3% (95% CI: 18.2–36.1%) of the coaches ended a season without being sacked. By mid-season, at round 19, the probability of coaches’ survival was 0.42 (95% CI: 0.32–0.53). Variation between season on survival estimates per round was substantial (between-season standard deviation = 0.48, 95% credible intervals: 0.25–0.95; corresponding to an inverse logit = 0.62, 95% CI: 0.56–0.72). Posterior estimates considering the influence of previous experience as head coach on the top Brazilian professional championship are presented as Supplementary Material (model m3). Conditional on the data, the observation of posterior draws posterior estimates for each coefficient of the interaction between each round and the proportion of novice. Experienced coaches showed that novice coaches had a similar survival probability to coaches with previous experience as head coach in the Brazilian top professional championship. As expected, there was substantial uncertainty in the proportion of experienced coaches across the 2012–2016 seasons. Codes, model summaries, and posterior predictive checks are available as Supplementary Material.

**Table 1:** Results from the Bayesian multilevel binomial model allowing for between-season variation at level 2.

Round	Estimate (95% credible interval)	Survival probability (95% credible interval), inverse logit estimate
1	3.62 (2.71–4.69)	0.97 (0.94–0.99)
2	2.64 (1.95–3.38)	0.93 (0.88–0.97)
3	1.93 (1.39–2.51)	0.87 (0.80–0.92)
4	1.61 (1.11–2.14)	0.83 (0.75–0.89)
5	1.25 (0.78–1.75)	0.78 (0.69–0.85)
6	0.95 (0.48–1.42)	0.72 (0.62–0.81)
7	0.68 (0.23–1.13)	0.66 (0.56–0.76)
8	0.64 (0.20–1.10)	0.65 (0.55–0.75)
9	0.57 (0.13–1.02)	0.64 (0.53–0.53)
10	0.50 (0.06–0.94)	0.62 (0.51–0.72)
11	0.35 (−0.08 to 0.79)	0.59 (0.48–0.69)
12	0.22 (−0.20 to 0.64)	0.55 (0.45–0.65)
13	0.15 (−0.30 to 0.60)	0.54 (0.43–0.65)
14	0.15 (−0.29 to 0.58)	0.54 (0.43–0.64)
15	0.08 (−0.34 to 0.51)	0.52 (0.42–0.62)
16	−0.05 (−0.47 to 0.36)	0.49 (0.38–0.59)
17	−0.12 (−0.55 to 0.31)	0.47 (0.37–0.58)
19	−0.32 (−0.75 to 0.11)	0.42 (0.32–0.53)
20	−0.36 (−0.79 to 0.06)	0.41 (0.31–0.51)
21	−0.39 (−0.82 to 0.03)	0.40 (0.31–0.51)
22	−0.39 (−0.82 to 0.05)	0.40 (0.31–0.51)
23	−0.43 (−0.88 to 0.01)	0.39 (0.20–0.50)
24	−0.49 (−0.93 to −0.04)	0.38 (0.28–0.49)
25	−0.60 (−1.04 to −0.16)	0.35 (0.26–0.46)
26	−0.71 (−1.15 to −0.27)	0.33 (0.24–0.43)
27	−0.79(−1.24 to −0.36)	0.31 (0.22–0.41)
28	−0.83 (−1.28 to −0.38)	0.30 (0.22–0.41)
29	−0.86 (−1.33 to −0.42)	0.30 (0.21–0.40)
30	−0.87 (−1.33 to −0.42)	0.30 (0.21–0.40)
31	−0.86 (−1.33 to −0.42)	0.30 (0.21–0.40)
32	−0.87 (−1.32 to −0.42)	0.30 (0.21–0.40)
33	−0.86 (−1.32 to −0.41)	0.30 (0.21–0.40)
34	−0.90 (−1.36 to −0.46)	0.29 (0.20–0.39)
35	−0.95 (−1.41 to −0.50)	0.28 (0.20–0.38)
36	−0.94 (−1.40 to −0.49)	0.28 (0.20–0.38)
37	−0.98 (−1.45 to −0.54)	0.27 (0.19–0.37)
38	−1.03 (−1.50 to −0.57)	0.26 (0.18–0.36)

## Discussion

To the best of our knowledge, this is the first study to consider data surrounding turnover of top South-American, in particular Brazilian, professional football coaches across several seasons. Hence, the probability of coaches’ survival in the top Brazilian professional football championship, considering variation across the competitive seasons between 2012 and 2017, was considered adopting a Bayesian multilevel framework. The survival probability of coaches in the top Brazilian professional football championship at the end of the seasons was about 26%. Hence, only about 7 coaches from 20 coaches at the beginning of each season were not dismissed during the season, indicating a high coaching turnover among the top Brazilian professional football championship. We also considered here the possibility that novice coaches (i.e., with no previous experience as a head coach in the Brazilian top professional championship) might differ from coaches with previous experience as head coach in the Brazilian top professional championship. Conditional on the data, the survival rates were consistent between coaches according to previous experience as a head coach experience in the Brazilian top professional championship.

Considering the coaches at the start of the season, the coach turnover in the Brazilian professional football championship between 2012 and 2017 was higher at the beginning of the seasons, and in particular, it was apparent that by the first quarter of the competitive season, about 40% of the coaches were already dismissed. The present results contrast with observations in European professional football leagues such as the Jupiler League in Belgium (Balduck et al., 2010), the Premier League in England (Bachan et al., 2008), the Bundesliga in Germany (Barros et al., 2009), the Serie A in Italy (Paola and Scoppa, 2012), the Eredivise in the Netherlands (van Ours and van Tuijl, 2016), or the La Liga in Spain (Lago-Peñas, 2011). These observations highlight the vulnerability of the professional football coach in the Brazilian top professional championship.

It has been shown in the first three divisions of the main football leagues in Belgium that after a coaching change, teams tended to have positive results in the first games with a new coach (Balduck et al., 2010). On the other hand, observations based on 14 season data in the Netherlands showed that coaches were retained even when results were less positive in the earlier games but showed an improvement in performance in the subsequent games (van Ours and van Tuijl, 2016). Furthermore, clubs that maintain the coaches during the whole season tend to present better results and tend to be ranked higher at the end of the season (Balduck et al., 2010; Lago-Peñas, 2011; Paola and Scoppa, 2012; van Ours and van Tuijl, 2016). On the other hand, there has been consistent observation in different contexts showing that coaching changes have either no effect on team performance, or a slight negative impact. Particularly in football data, a null effect of coaching changes on team performance has been demonstrated in the Italian league (Paola and Scoppa, 2012) and the Dutch Premier League (Koning, 2003). Moreover, there are available observations that coaching changes resulted in worse team performance in English professional football (Audas et al., 1997). Similar null or negative trends between coaching changes and null or negative team performance have been noted in NBA teams (Giambatista, 2004), NFL (Rowe et al., 2005) and college basketball (Fizel and D’Itri, 1999). Hence, Brazilian club boards appear to overvalue the short-term impact of a coaching change, likely disregarding and/or compromising the potential for coaches to fully undertake their task. However, the coaches’ professional context is complex and dynamic, hence other factors other than winning may contribute to maintain or dismiss the coach in the top Brazilian teams. Often, the power of the individuals involved in the club management, ownership and coach tenure, as well as expectations and the coach’s reputation, all together contributes to alter the likelihood of dismissal (Wangrow et al., 2018). For example, considering other professional sports, observations in the NBA showed that first-year coaches had an important rate of dismissal, about 16% of the coaches (Wangrow et al., 2018).

Particularly in the present results, coaches were more likely to be dismissed during the first seven games, where about 35% of the coaches had been dismissed, on average, across the seasons. These results contrast with the observation in European leagues, where coaches’ turnover tends to occur later in the season (Bachan et al., 2008; van Ours and van Tuijl, 2016). Overall, the present results showed a high probability (74%) of coaches being dismissed across the 38-round season, accounting for season variation in the top professional Brazilian football championship. Again, the present results markedly contrast with observations in European top football leagues. For example, in the Netherlands, it has been observed that after 34 games, 15–20% of coaches’ changes were caused by dismissal, while 10% of coaches apparently left the club voluntarily (van Ours and van Tuijl, 2016).

The comparison between the un-pooled binary model, based on aggregated binomial model without accounting for variation between season, and the partial pooled model based on multilevel binomial model highlighted the importance of clustering effects on the data. The need to consider the influence of group- or macro-level variables has been noted in other areas (Diez-Roux, 2000; Goldstein, 2011; McElreath, 2015), particularly in longitudinal observations such as event history analysis (Singer and Willett, 2003). Although there are studies considering multilevel approaches, particularly modeling coaches’ turnover (Barros et al., 2009), its use in sports and exercise research is still limited. The present study adds by illustrating the application of a simple binomial modeling to describe the probability of coaches’ survival across the season, allowing for variation between higher units of observation, in the present case’ the multiple seasons observed. We also illustrated the possibility to consider covariates to explore between the coaches’ variation in each round and across the period of observation. Furthermore, we used Bayesian methods to fit the models as an alternative to the limitations of frequentist methods (Gelman and Shalizi, 2013), as well as other approaches used in sports and exercise science (Welsh and Knight, 2015; Sainani, 2018). In particular, Bayesian methods consider parameters as random variables combining both sample data and prior distribution information to estimate posterior information (Gelman et al., 2013; McElreath, 2015). Bayesian estimations allow a probabilistic interpretation of how different parameters are used to simulate predictions and assess the quality and fit of the model (McElreath and Koster, 2014). Although we were not able to retain reliable information about coaches’ characteristics that would allow a deeper understanding of the overall coaching turnover in the top Brazilian Football Championship within and between seasons, we illustrate an analytical framework to describe an applied example of time-event data in exercise and sport sciences.

In the Supplementary Material, we provide additional information about how we modeled the data, specifically the codes and assessment of the quality and fit of the derived models.

In the present study, we considered the available data of the Brazilian Football Confederation, which did not enable us to explore reasons for coaches’ job termination during the period of observation (e.g., dismissal, voluntary exit, transfer to another team, retirement, among others). Also, we only considered coaches who started the season, which likely underestimates the overall coaching turnover in the top Brazilian Football Championship within and between seasons.

In summary, there was a substantial turnover of coaches in the Brazilian Football Championship between 2012 and 2017, with a large amount of variation between seasons. The present results expose the vulnerability of the coaching context in Brazilian football, apparently independent of the coaches’ previous experience as head coach in the Brazilian Football Championship, potentially highlighting an excessive emphasis on short-term results to mediate club management decisions.

## Data Availability

The datasets generated for this study are available on request to the corresponding author.

## Author Contributions

AT, FM, RR, and WS contributed conception and design of the study. AT, FM, HC, RR, and WS organized the database. HC performed the statistical analysis. AT, FM, RR, and WS wrote the first drafts of the manuscript. AT, FM, HC, JN, and MM wrote sections of the manuscript. All authors contributed to manuscript review, read, and approved the submitted version.

### Conflict of Interest Statement

The authors declare that the research was conducted in the absence of any commercial or financial relationships that could be construed as a potential conflict of interest.

## References

[ref1] Audas R. Dobson S. Goddard J. (1997). Team performance and managerial change in the English Football League. Econ. Aff. 17, 30–36. 10.1111/1468-0270.00039

[ref2] Bachan R. Reilly B. Witt R. (2008). The hazard of being an English football league manager: empirical estimates for three recent league seasons. J. Oper. Res. Soc. 59, 884–891. 10.1057/palgrave.jors.2602408

[ref3] Balduck A.-L. Buelens M. Philippaerts R. (2010). Short-term effects of midseason coach turnover on team performance in soccer. Res. Q. Exerc. Sport 81, 379–383. 10.1080/02701367.2010.10599686, PMID: 20949858

[ref4] Barros C. P. Frick B. Passos J. (2009). Coaching for survival: the hazards of head coach careers in the German “Bundesliga”. Appl. Econ. 41, 3303–3311. 10.1080/00036840701721455

[ref5] Bell A. Brooks C. Markham T. (2013). The performance of football club managers: skill or luck? Econ. Financ. Res. 1, 19–30. 10.1080/21649480.2013.768829

[ref6] Brazil (1993). Law no 8650. It deals with the work relations of the professional football coach and gives other measures. (Brasília, DF: Diário oficial da União). (Official journal of State).

[ref501] Bürkner P. C. (2017). brms: An R package for bayesian generalized linear mixed models using Stan. J. Stat. Softw. 80, 1–28. 10.18637/jss.v080.i01

[ref7] Diez-Roux A. V. (2000). Multilevel analysis in public health research. Annu. Rev. Public Health 21, 171–192. 10.1146/annurev.publhealth.21.1.171, PMID: 10884951

[ref8] Duffy P. Hartley H. Bales J. Crespo M. Dick F. Vardhan D. (2011). Sport coaching as a “profession”: challenges and future directions. Intern. J. Coach. Sci. 5, 93–123.

[ref9] Fifa.Com (2019). *FIFA/Coca-Cola World Ranking* Available at: https://www.fifa.com/fifa-world-ranking/associations/association=bra/men/index.html (Accessed February 07, 2019).

[ref10] Fizel J. L. D’itri M. P. (1999). Firing and hiring of managers: does efficiency matter? J. Manag. 25, 567–585.

[ref11] Gelman A. Carlin J. B. Stern H. S. Dunson D. B. Vehtari A. Rubin D. B. (2013). Bayesian data analysis, 3rd edn. (Boca Raton, FL: Chapman & Hall/CRC Press).

[ref12] Gelman A. Hill J. (2007). Data analysis using regression and multilevel/hierarchical models. (Cambridge: Cambridge University Press).

[ref13] Gelman A. Shalizi C. R. (2013). Philosophy and the practice of Bayesian statistics. Br. J. Math. Stat. Psychol. 66, 8–38. 10.1111/j.2044-8317.2011.02037.x, PMID: 22364575 PMC4476974

[ref14] Giambatista R. C. (2004). Jumping through hoops: a longitudinal study of leader life cycles in the NBA. Leadersh. Q. 15, 607–624. 10.1016/j.leaqua.2004.07.002

[ref15] Goldstein H. (2011). Multilevel statistical models. (Chichester, West Sussex: Wiley).

[ref16] Hoffmann R. Ging L. C. Ramasamy B. (2002). The socio-economic determinants of international soccer performance. J. Appl. Econ. 5, 253–272. 10.1080/15140326.2002.12040579

[ref17] Koning R. H. (2003). An econometric evaluation of the effect of firing a coach on team performance. Appl. Econ. 35, 555–564. 10.1080/0003684022000015946

[ref18] Lago-Peñas C. (2011). Coach mid-season replacement and team performance in professional soccer. J. Hum. Kinet. 28, 115–122. 10.2478/v10078-011-0028-7, PMID: 23487177 PMC3592111

[ref19] Lyle J. (2002). Sports coaching concepts: A framework for coaches' behaviour. (London: Routledge).

[ref20] Mallett C. J. Lara-Bercial S. (2016). “Serial winning coaches: people, vision, and environment” in Sport and exercise psychology research: From theory to practice. eds. Raab M. Wylleman P. Seiler R. Elbe A. Hatzigeorgiadis A. (Amsterdam, The Netherlands: Elsevier), 289–322. 10.1016/B978-0-12-803634-1.00014-5

[ref21] Mallett C. Rynne S. (2015). “Changing role of coaches across development” in Routledge handbook of sport expertise. ed. Damian J. F. B. (Abingdon: Routledge).

[ref22] McElreath R. (2015). Statistical rethinking: A Bayesian course with examples in R and Stan. (Boca Raton, FL: Chapman & Hall/CRC Press).

[ref23] McElreath R. Koster J. (2014). Using multilevel models to estimate variation in foraging returns. Effects of failure rate, harvest size, age, and individual heterogeneity. Hum. Nat. 25, 100–120. 10.1007/s12110-014-9193-4, PMID: 24522975

[ref24] Paola M. D. Scoppa V. (2012). The effects of managerial turnover: evidence from coach dismissals in Italian soccer teams. J. Sports Econ. 13, 152–168. 10.1177/1527002511402155

[ref25] Rowe W. G. Cannella A. A. Rankin D. Gorman D. (2005). Leader succession and organizational performance: integrating the common-sense, ritual scapegoating, and vicious-circle succession theories. Leadersh. Q. 16, 197–219. 10.1016/j.leaqua.2005.01.001

[ref26] Sainani K. L. (2018). The problem with “magnitude-based inference”. Med. Sci. Sports Exerc. 50, 2166–2176. 10.1249/MSS.0000000000001645, PMID: 29683920

[ref27] Singer J. D. Willett J. B. (2003). Applied longitudinal data analysis: Modeling change and event occurrence. (Oxford; New York: Oxford University Press).

[ref500] Stan Development Team (2015). Stan: A C++ Library for Probability and Sampling, Version 2.7.0. http://mc-stan.org/

[ref28] Steele F. (2011). Multilevel discrete-time event history models with applications to the analysis of recurrent employment transitions. Aust. N. Z. J. Stat. 53, 1–20. 10.1111/j.1467-842X.2011.00604.x

[ref29] van Ours J. C. Van Tuijl M. A. (2016). In-season head-coach dismissals and the performance of professional football teams. Econ. Inq. 54, 591–604. 10.1111/ecin.12280

[ref30] Vehtari A. Gelman A. Gabry J. (2016). Practical Bayesian model evaluation using leave-one-out cross-validation and WAIC. ArXiv e-prints [Online]. 27, 1413–1432. Available at: http://arxiv.org/abs/1507.04544

[ref31] Wangrow D. B. Schepker D. J. Barker Iii V. L. (2018). Power, performance, and expectations in the dismissal of NBA coaches: a survival analysis study. Sport Manag. Rev. 21, 333–346. 10.1016/j.smr.2017.08.002

[ref32] Welsh A. H. Knight E. J. (2015). “Magnitude-based inference”: a statistical review. Med. Sci. Sports Exerc. 47, 874–884. 10.1249/MSS.0000000000000451, PMID: 25051387 PMC5642352

