# Supplementary material for: Coach Turnover in Top Professional Brazilian Football Championship: A Multilevel Survival Analysis
Source: Front Psychol. 2019 Jun 6;10:1246. doi: 10.3389/fpsyg.2019.01246 (PMC6562306; doi:10.3389/fpsyg.2019.01246)

Supplementary material for the manuscript “Coach turnover in top professional Brazilian football championship: a multilevel survival analysis"

Authors: Alexandre B. Tozetto, Humberto M. Carvalho, Rodolfo S. Rosa, Felipe G. Mendes, Walan R. Silva, Juarez V. Nascimento, Michel Milistetd

R code, model summary, trace plot and posterior predictive check for un-pooled binomial regression model to describe survival probability for the coaches in the top professional Brazilian football championship between 2012 and 2017.

#load brms package

library(brms)

Loading required package: Rcpp

Loading required package: ggplot2

Loading 'brms' package (version 2.4.0). Useful instructions

can be found by typing help('brms'). A more detailed introduction

to the package is available through vignette('brms_overview').

Run theme_set(theme_default()) to use the default bayesplot theme.

#un-pooled binomial regression model code

m1<-brm(data = coach3, family = binomial,

+ surv | trials(density) ~ 0 + factor(round),

+ prior(normal(0, 1), class = b),

+ chains = 2, iter = 4000, warmup = 1000, cores = 4,

+ control = list(adapt_delta=0.9))

#model summary

summary(m1)

Family: binomial

Links: mu = logit

Formula: surv | trials(density) ~ 0 + factor(round)

Data: coach3 (Number of observations: 228)

Samples: 2 chains, each with iter = 4000; warmup = 1000; thin = 1;

total post-warmup samples = 6000

Population-Level Effects:

Estimate Est.Error l-95% CI u-95% CI Eff.Sample Rhat

factorround1 3.58 0.48 2.73 4.58 6000 1.00

factorround2 2.61 0.34 1.98 3.32 6000 1.00

factorround3 1.90 0.27 1.40 2.43 6000 1.00

factorround4 1.59 0.24 1.13 2.10 6000 1.00

factorround5 1.24 0.21 0.84 1.66 6000 1.00

factorround6 0.94 0.20 0.56 1.34 6000 1.00

factorround7 0.67 0.19 0.31 1.05 6000 1.00

factorround8 0.64 0.19 0.28 1.01 6000 1.00

factorround9 0.57 0.19 0.20 0.95 6000 1.00

factorround10 0.50 0.18 0.14 0.86 6000 1.00

factorround11 0.36 0.18 0.01 0.73 6000 1.00

factorround12 0.23 0.18 -0.13 0.59 6000 1.00

factorround13 0.17 0.18 -0.20 0.52 6000 1.00

factorround14 0.16 0.18 -0.19 0.51 6000 1.00

factorround15 0.10 0.18 -0.26 0.44 6000 1.00

factorround16 -0.03 0.17 -0.38 0.31 6000 1.00

factorround17 -0.10 0.18 -0.46 0.25 6000 1.00

factorround18 -0.13 0.18 -0.48 0.22 6000 1.00

factorround19 -0.29 0.18 -0.65 0.06 6000 1.00

factorround20 -0.33 0.18 -0.70 0.03 6000 1.00

factorround21 -0.36 0.18 -0.72 -0.02 6000 1.00

factorround22 -0.36 0.18 -0.71 -0.00 6000 1.00

factorround23 -0.39 0.18 -0.75 -0.04 6000 1.00

factorround24 -0.46 0.18 -0.82 -0.11 6000 1.00

factorround25 -0.57 0.18 -0.94 -0.21 6000 1.00

factorround26 -0.67 0.19 -1.04 -0.31 6000 1.00

factorround27 -0.75 0.19 -1.13 -0.37 6000 1.00

factorround28 -0.79 0.19 -1.17 -0.41 6000 1.00

factorround29 -0.82 0.19 -1.22 -0.46 6000 1.00

factorround30 -0.82 0.19 -1.22 -0.45 6000 1.00

factorround31 -0.82 0.20 -1.21 -0.45 6000 1.00

factorround32 -0.82 0.19 -1.21 -0.46 6000 1.00

factorround33 -0.82 0.19 -1.21 -0.45 6000 1.00

factorround34 -0.86 0.19 -1.25 -0.49 6000 1.00

factorround35 -0.90 0.20 -1.30 -0.51 6000 1.00

factorround36 -0.90 0.20 -1.29 -0.51 6000 1.00

factorround37 -0.94 0.20 -1.34 -0.56 6000 1.00

factorround38 -0.98 0.20 -1.39 -0.59 6000 1.00

Samples were drawn using sampling(NUTS). For each parameter, Eff.Sample

is a crude measure of effective sample size, and Rhat is the potential

scale reduction factor on split chains (at convergence, Rhat = 1).

#trace plot

plot(m1)


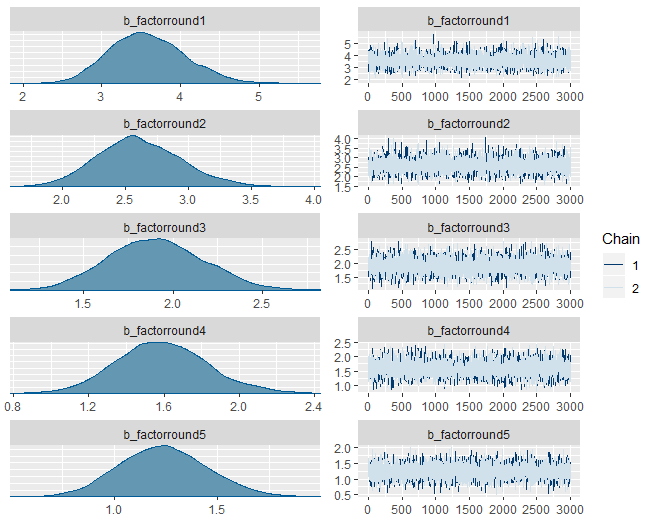


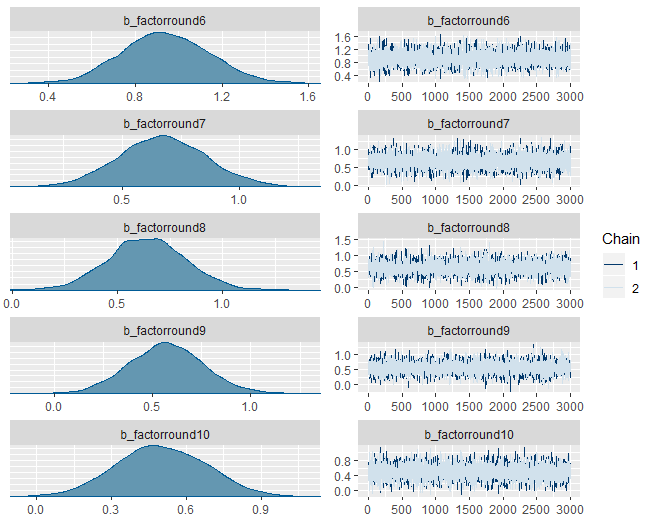


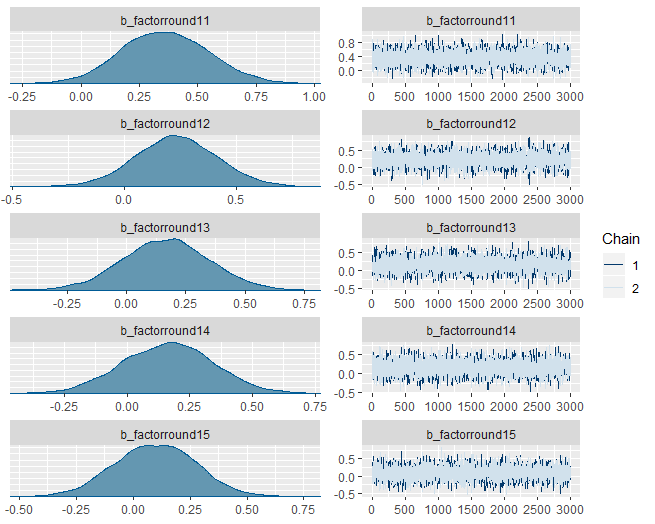


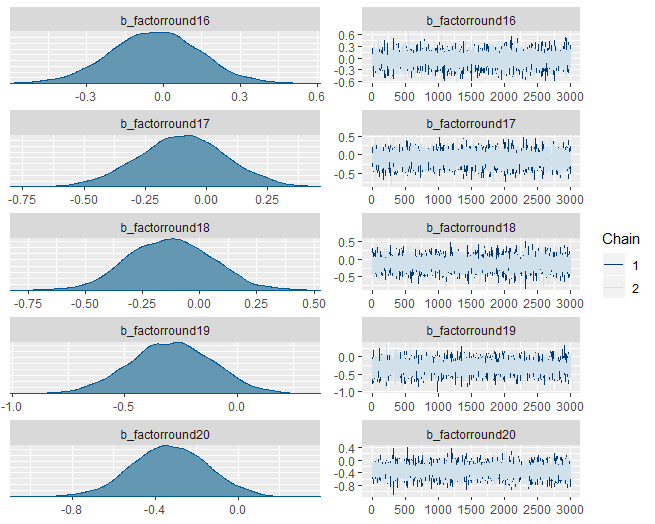


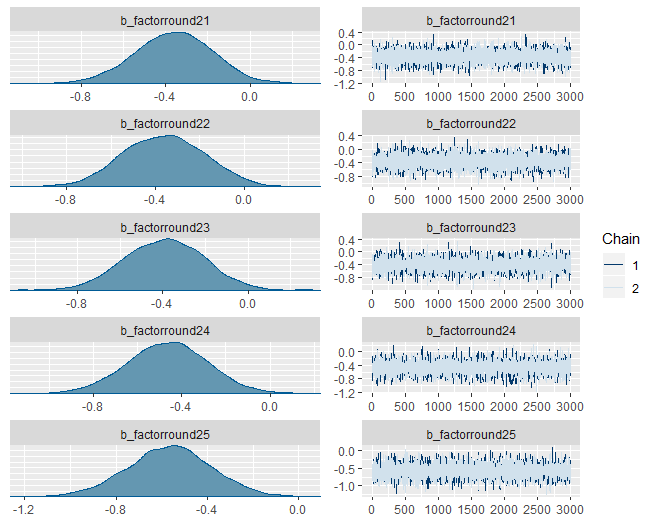


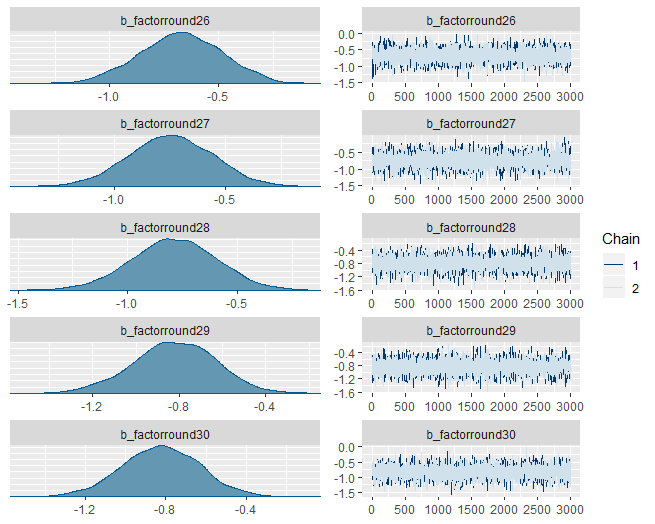


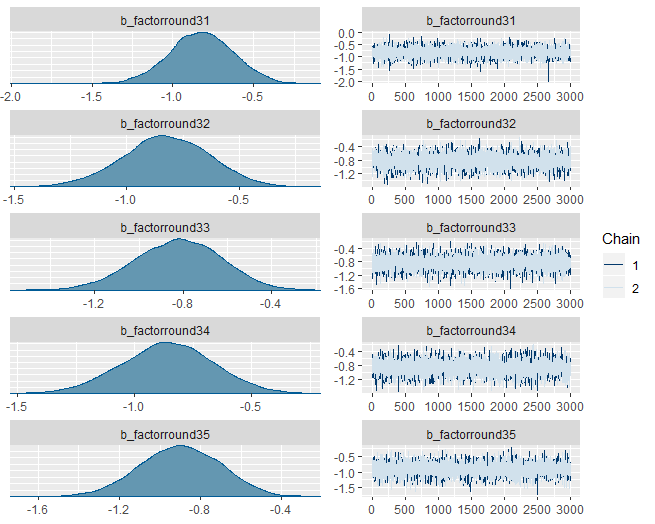


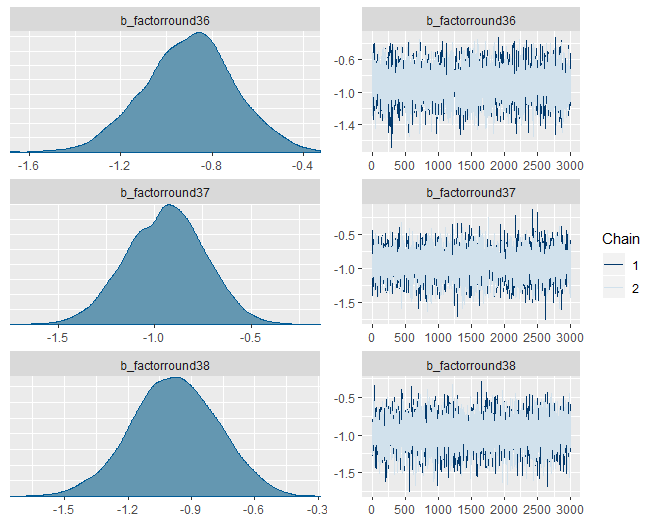


#posterior predictive checks

pp_check(m1,nsamples = 100)


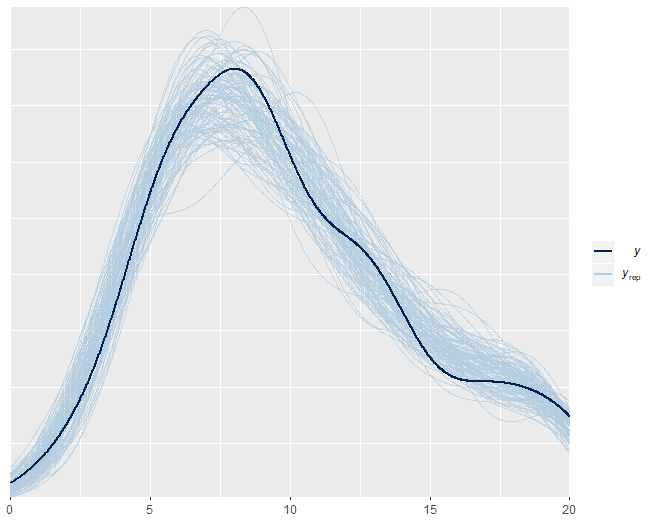


R code, model summary, trace plot and posterior predictive check for partial-pooled logistic regression model to describe survival probability for the coaches in the top professional Brazilian football championship between 2012 and 2017. In this model we allow for survival probability to vary by season at level-2.

#multilevel model code

m2<-brm(data = coach3, family = binomial,

+ surv | trials(density) ~ factor(round)+0 + (1 | season),

+ prior = c(set_prior("normal(0, 1)", class = "b"),

+ ("cauchy(0, 1)", class = "sd")),

+ chains = 2, iter = 4000, warmup = 1000, cores = 4,

+ control = list(adapt_delta=0.9))

#model summary

summary(m2)

Family: binomial

Links: mu = logit

Formula: surv | trials(density) ~ factor(round) + 0 + (1 | season)

Data: coach3 (Number of observations: 228)

Samples: 2 chains, each with iter = 4000; warmup = 1000; thin = 1;

total post-warmup samples = 6000

Group-Level Effects:

~season (Number of levels: 6)

Estimate Est.Error l-95% CI u-95% CI Eff.Sample Rhat

sd(Intercept) 0.47 0.17 0.25 0.87 1952 1.00

Population-Level Effects:

Estimate Est.Error l-95% CI u-95% CI Eff.Sample Rhat

factorround1 3.62 0.50 2.70 4.66 6000 1.00

factorround2 2.63 0.36 1.96 3.39 6000 1.00

factorround3 1.92 0.28 1.39 2.48 6000 1.00

factorround4 1.61 0.27 1.10 2.16 6000 1.00

factorround5 1.25 0.25 0.78 1.74 6000 1.00

factorround6 0.95 0.23 0.50 1.40 6000 1.00

factorround7 0.68 0.23 0.24 1.11 6000 1.00

factorround8 0.64 0.23 0.20 1.07 6000 1.00

factorround9 0.57 0.22 0.13 1.01 6000 1.00

factorround10 0.49 0.22 0.06 0.92 2068 1.00

factorround11 0.35 0.22 -0.09 0.77 1986 1.00

factorround12 0.21 0.21 -0.20 0.64 2124 1.00

factorround13 0.15 0.21 -0.28 0.57 6000 1.00

factorround14 0.15 0.22 -0.28 0.59 6000 1.00

factorround15 0.08 0.22 -0.33 0.51 6000 1.00

factorround16 -0.06 0.22 -0.48 0.36 6000 1.00

factorround17 -0.12 0.22 -0.55 0.31 2141 1.00

factorround18 -0.15 0.22 -0.58 0.27 6000 1.00

factorround19 -0.33 0.21 -0.75 0.09 2182 1.00

factorround20 -0.36 0.22 -0.79 0.06 6000 1.00

factorround21 -0.39 0.22 -0.83 0.03 6000 1.00

factorround22 -0.39 0.22 -0.83 0.02 6000 1.00

factorround23 -0.43 0.22 -0.86 -0.01 6000 1.00

factorround24 -0.50 0.23 -0.94 -0.05 6000 1.00

factorround25 -0.61 0.22 -1.05 -0.18 6000 1.00

factorround26 -0.71 0.23 -1.15 -0.27 6000 1.00

factorround27 -0.79 0.23 -1.24 -0.34 6000 1.00

factorround28 -0.83 0.23 -1.28 -0.39 6000 1.00

factorround29 -0.87 0.23 -1.32 -0.43 6000 1.00

factorround30 -0.87 0.23 -1.33 -0.42 6000 1.00

factorround31 -0.87 0.23 -1.33 -0.42 6000 1.00

factorround32 -0.87 0.23 -1.31 -0.42 6000 1.00

factorround33 -0.87 0.23 -1.32 -0.42 6000 1.00

factorround34 -0.91 0.23 -1.37 -0.46 6000 1.00

factorround35 -0.95 0.23 -1.42 -0.50 6000 1.00

factorround36 -0.95 0.23 -1.41 -0.50 6000 1.00

factorround37 -0.99 0.24 -1.46 -0.53 6000 1.00

factorround38 -1.03 0.23 -1.49 -0.58 6000 1.00

Samples were drawn using sampling(NUTS). For each parameter, Eff.Sample

is a crude measure of effective sample size, and Rhat is the potential

scale reduction factor on split chains (at convergence, Rhat = 1).

#trace plot

plot(m2)


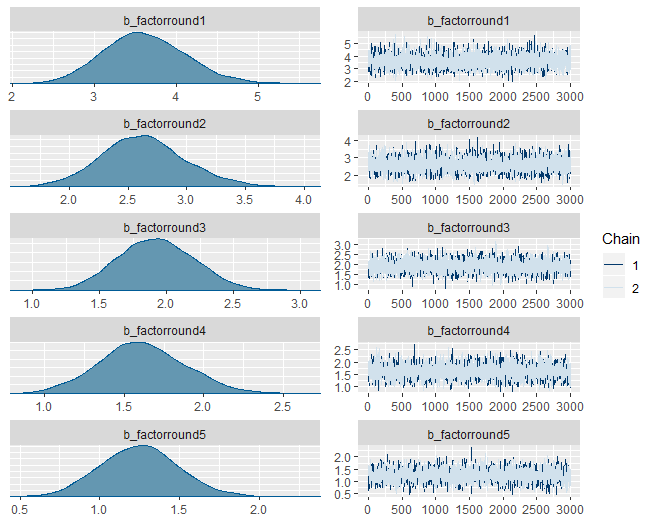


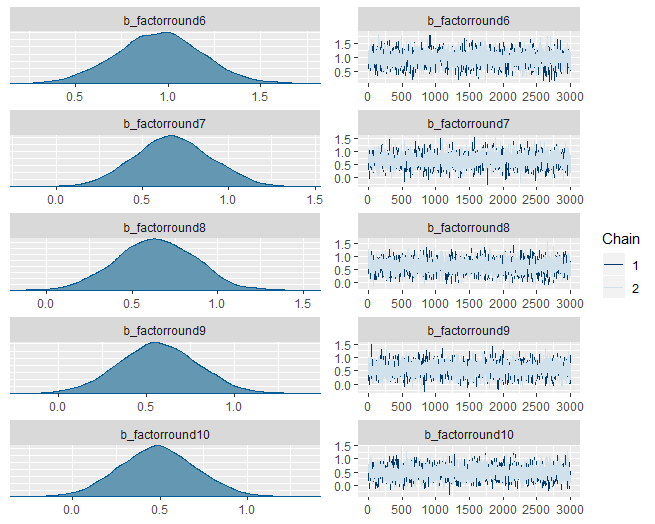


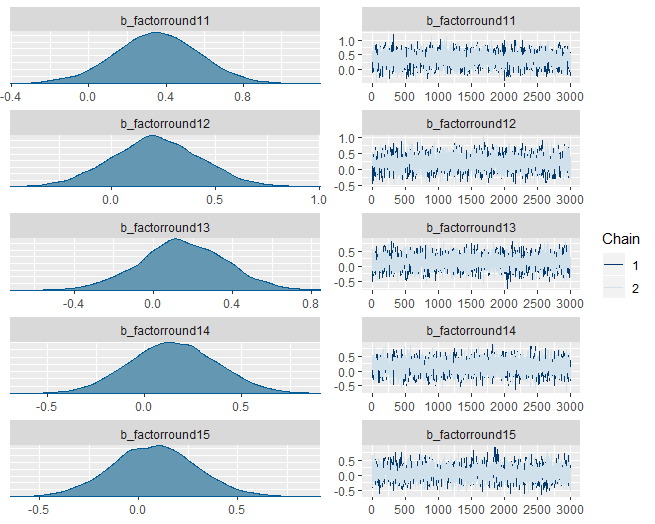


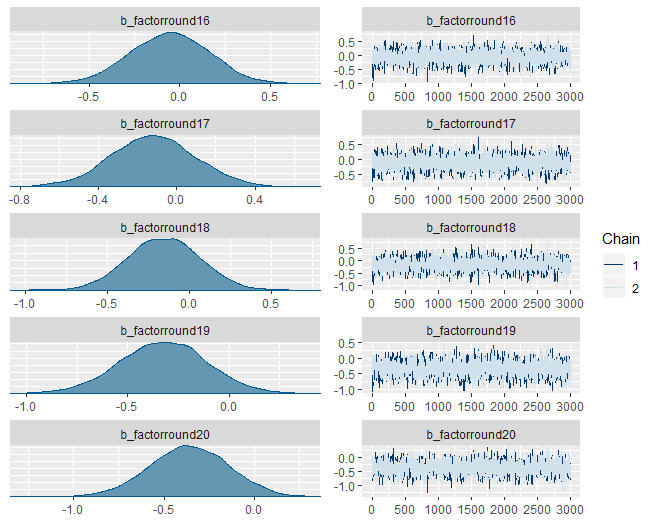


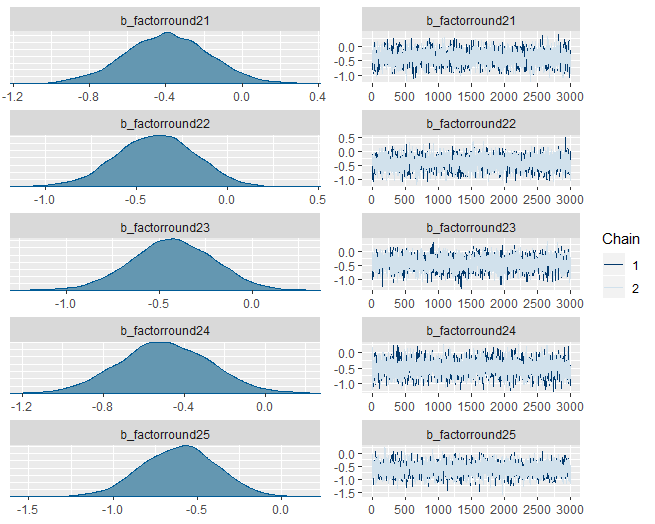


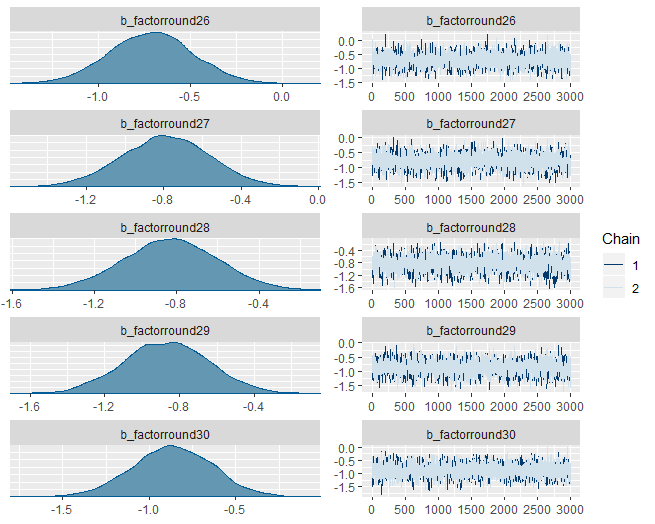


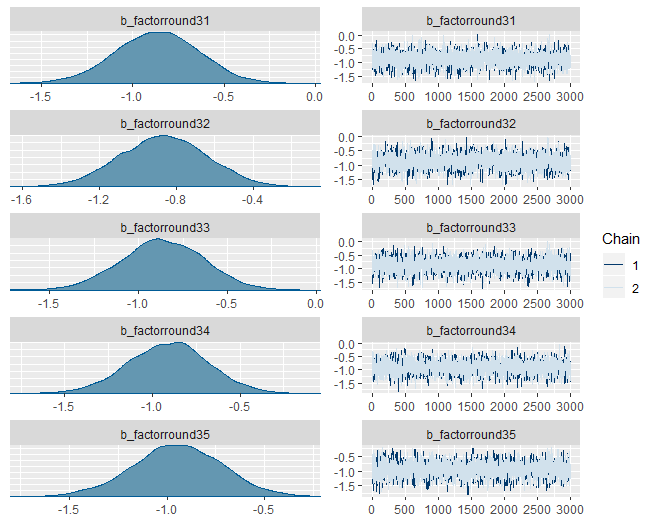


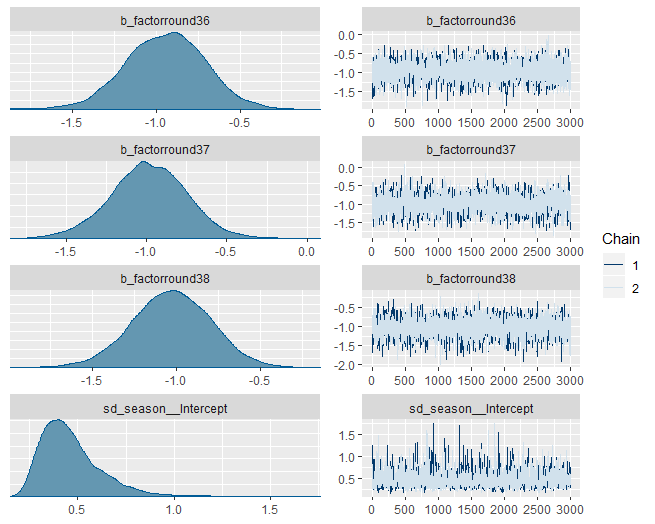


#posterior predictive checks

pp_check(m2,nsamples = 100)


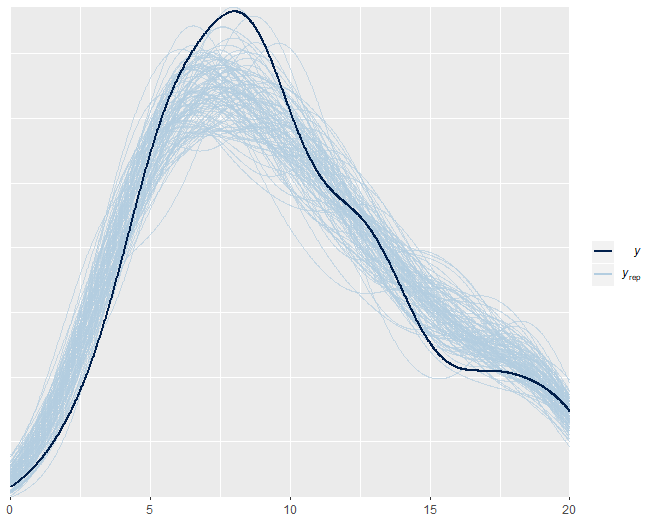


Comparison between un-pooled model (m1) and multilevel model (m2)

waic(m1,m2)

WAIC SE

m1 998.30 21.60

m2 848.82 9.79

m1 - m2 149.48 18.44

#multilevel model considering coaches proportion as population-level level effects in each round, allowing to vary between seasons at level-2

m3<-brm(data = coach3, family = binomial,

+ surv | trials(density) ~ factor(round)*coach_proportion+0+(coach_proportion | season),

+ prior = c(set_prior("normal(0, 1)", class = "b"),

+ ("cauchy(0, 1)", class = "sd")),

+ chains = 2, iter = 4000, warmup = 1000, cores = 4,

+ control = list(adapt_delta=0.99,max_treedepth=15))

#model summary

summary(m3)

Family: binomial

Links: mu = logit

Formula: surv | trials(density) ~ factor(round) * coach_proportion + 0 + (coach_proportion | season)

Data: coach3 (Number of observations: 228)

Samples: 2 chains, each with iter = 4000; warmup = 1000; thin = 1;

total post-warmup samples = 6000

Group-Level Effects:

~season (Number of levels: 6)

Estimate Est.Error l-95% CI u-95% CI

sd(Intercept) 0.68 0.52 0.03 1.97

sd(coach_proportion) 1.01 0.61 0.16 2.57

cor(Intercept,coach_proportion) -0.55 0.51 -1.00 0.81

Eff.Sample Rhat

sd(Intercept) 3125 1.00

sd(coach_proportion) 2107 1.00

cor(Intercept,coach_proportion) 1660 1.00

Population-Level Effects:

Estimate Est.Error l-95% CI u-95% CI Eff.Sample Rhat

factorround1 3.60 0.51 2.66 4.62 6000 1.00

factorround2 1.77 0.70 0.43 3.15 6000 1.00

factorround3 1.30 0.68 -0.06 2.62 6000 1.00

factorround4 1.07 0.65 -0.21 2.39 6000 1.00

factorround5 0.82 0.67 -0.46 2.11 6000 1.00

factorround6 0.70 0.65 -0.54 2.00 6000 1.00

factorround7 0.49 0.68 -0.84 1.78 6000 1.00

factorround8 0.48 0.67 -0.83 1.80 6000 1.00

factorround9 0.29 0.65 -0.98 1.52 6000 1.00

factorround10 0.25 0.65 -1.03 1.51 6000 1.00

factorround11 0.32 0.65 -0.94 1.59 6000 1.00

factorround12 0.20 0.64 -1.05 1.46 6000 1.00

factorround13 0.25 0.66 -1.10 1.53 6000 1.00

factorround14 0.24 0.68 -1.09 1.61 6000 1.00

factorround15 0.21 0.67 -1.07 1.49 6000 1.00

factorround16 0.03 0.69 -1.30 1.38 6000 1.00

factorround17 -0.08 0.69 -1.40 1.23 6000 1.00

factorround18 -0.07 0.67 -1.40 1.25 6000 1.00

factorround19 -0.23 0.67 -1.55 1.07 6000 1.00

factorround20 -0.26 0.66 -1.56 1.02 6000 1.00

factorround21 -0.30 0.65 -1.57 0.99 6000 1.00

factorround22 -0.29 0.66 -1.58 1.02 6000 1.00

factorround23 -0.27 0.65 -1.56 1.02 6000 1.00

factorround24 -0.30 0.65 -1.55 0.98 6000 1.00

factorround25 -0.36 0.65 -1.61 0.92 6000 1.00

factorround26 -0.48 0.65 -1.77 0.79 6000 1.00

factorround27 -0.61 0.66 -1.91 0.71 6000 1.00

factorround28 -0.65 0.64 -1.91 0.57 6000 1.00

factorround29 -0.53 0.66 -1.82 0.78 6000 1.00

factorround30 -0.53 0.67 -1.86 0.77 6000 1.00

factorround31 -0.54 0.67 -1.84 0.77 6000 1.00

factorround32 -0.53 0.67 -1.84 0.76 6000 1.00

factorround33 -0.54 0.67 -1.85 0.72 6000 1.00

factorround34 -0.53 0.67 -1.80 0.78 6000 1.00

factorround35 -0.53 0.68 -1.85 0.79 6000 1.00

factorround36 -0.54 0.68 -1.85 0.80 6000 1.00

factorround37 -0.52 0.68 -1.89 0.79 6000 1.00

factorround38 -0.52 0.66 -1.85 0.74 6000 1.00

coach_proportion -0.01 0.37 -0.79 0.67 4459 1.00

factorround2:coach_proportion 1.14 0.79 -0.34 2.65 6000 1.00

factorround3:coach_proportion 0.76 0.78 -0.75 2.29 6000 1.00

factorround4:coach_proportion 0.65 0.74 -0.80 2.08 6000 1.00

factorround5:coach_proportion 0.48 0.74 -0.98 1.94 6000 1.00

factorround6:coach_proportion 0.26 0.73 -1.17 1.67 6000 1.00

factorround7:coach_proportion 0.17 0.74 -1.27 1.61 6000 1.00

factorround8:coach_proportion 0.15 0.73 -1.31 1.56 6000 1.00

factorround9:coach_proportion 0.28 0.71 -1.08 1.67 6000 1.00

factorround10:coach_proportion 0.23 0.71 -1.15 1.63 6000 1.00

factorround11:coach_proportion -0.00 0.72 -1.42 1.41 6000 1.00

factorround12:coach_proportion -0.02 0.71 -1.42 1.35 6000 1.00

factorround13:coach_proportion -0.15 0.73 -1.56 1.30 6000 1.00

factorround14:coach_proportion -0.14 0.74 -1.60 1.30 6000 1.00

factorround15:coach_proportion -0.18 0.72 -1.56 1.26 6000 1.00

factorround16:coach_proportion -0.13 0.75 -1.60 1.33 6000 1.00

factorround17:coach_proportion -0.07 0.74 -1.52 1.40 6000 1.00

factorround18:coach_proportion -0.12 0.72 -1.56 1.32 6000 1.00

factorround19:coach_proportion -0.14 0.73 -1.57 1.30 6000 1.00

factorround20:coach_proportion -0.15 0.72 -1.53 1.28 6000 1.00

factorround21:coach_proportion -0.14 0.71 -1.57 1.21 6000 1.00

factorround22:coach_proportion -0.14 0.72 -1.59 1.27 6000 1.00

factorround23:coach_proportion -0.21 0.72 -1.62 1.19 6000 1.00

factorround24:coach_proportion -0.26 0.71 -1.63 1.12 6000 1.00

factorround25:coach_proportion -0.32 0.72 -1.74 1.08 6000 1.00

factorround26:coach_proportion -0.32 0.73 -1.78 1.13 6000 1.00

factorround27:coach_proportion -0.25 0.71 -1.66 1.15 6000 1.00

factorround28:coach_proportion -0.25 0.69 -1.63 1.12 6000 1.00

factorround29:coach_proportion -0.36 0.71 -1.74 1.00 6000 1.00

factorround30:coach_proportion -0.37 0.71 -1.74 1.03 6000 1.00

factorround31:coach_proportion -0.36 0.71 -1.75 1.04 6000 1.00

factorround32:coach_proportion -0.37 0.70 -1.74 0.99 6000 1.00

factorround33:coach_proportion -0.36 0.71 -1.72 1.08 6000 1.00

factorround34:coach_proportion -0.41 0.71 -1.82 0.95 6000 1.00

factorround35:coach_proportion -0.46 0.71 -1.85 0.96 6000 1.00

factorround36:coach_proportion -0.45 0.72 -1.89 0.95 6000 1.00

factorround37:coach_proportion -0.51 0.73 -1.90 0.93 6000 1.00

factorround38:coach_proportion -0.56 0.71 -1.93 0.83 6000 1.00

Samples were drawn using sampling(NUTS). For each parameter, Eff.Sample

is a crude measure of effective sample size, and Rhat is the potential

scale reduction factor on split chains (at convergence, Rhat = 1).

#trace plot

plot(m3)


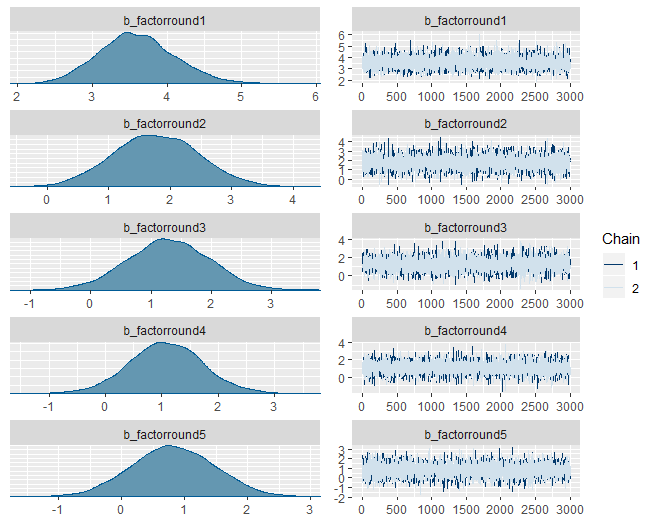


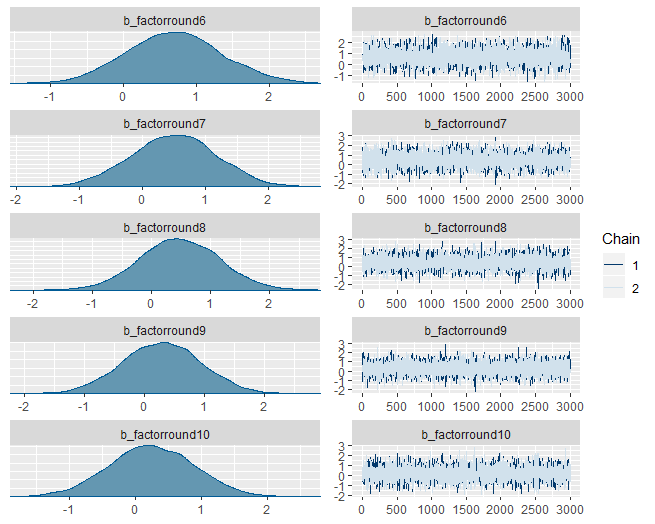


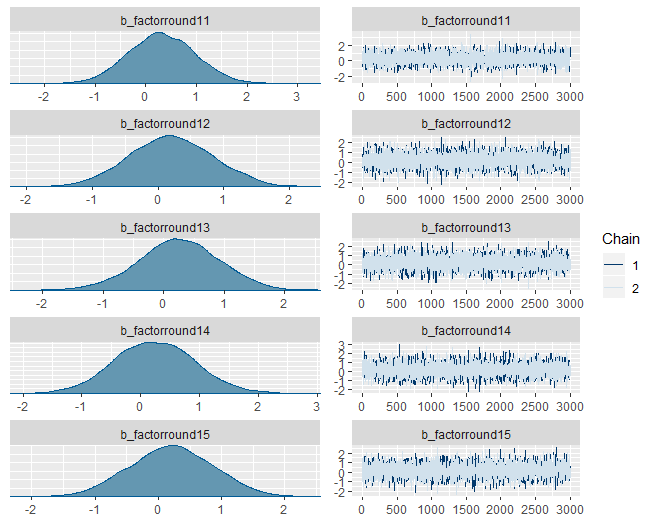


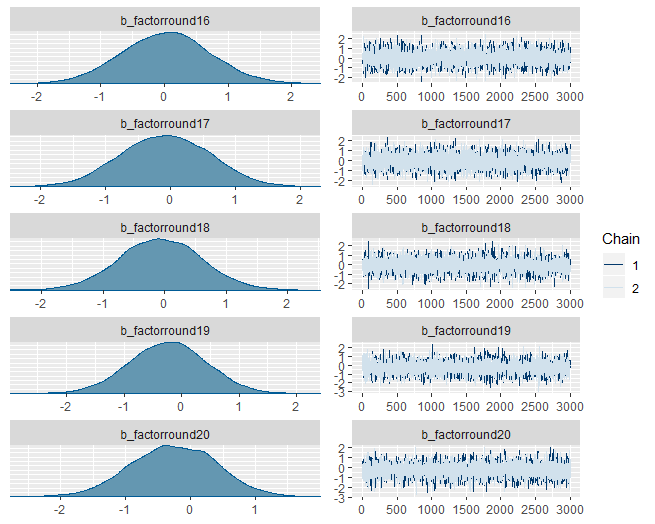


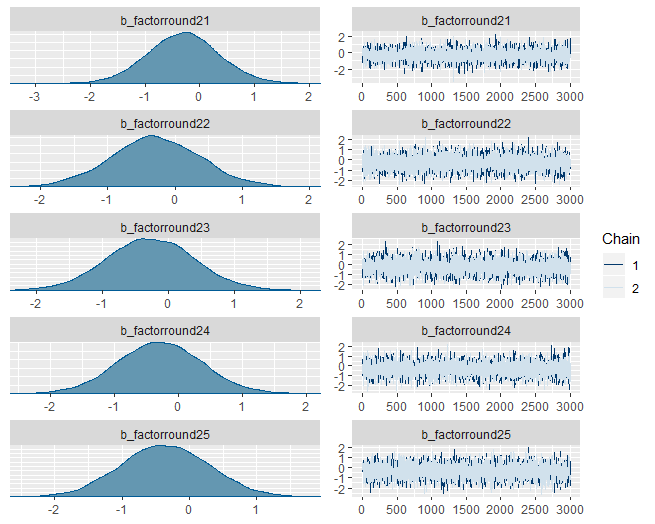


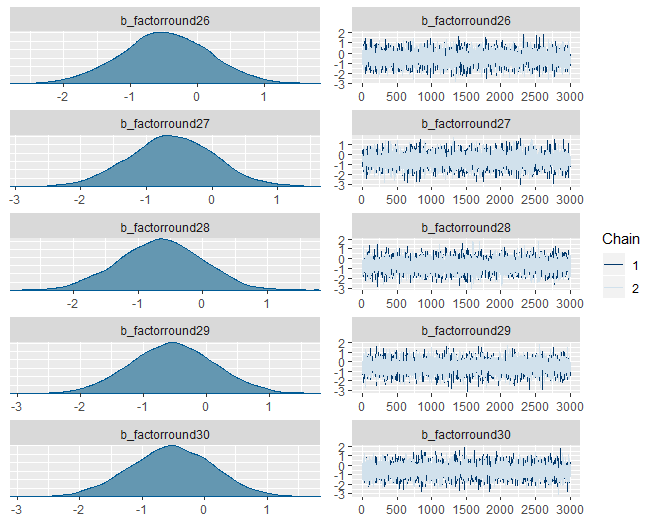


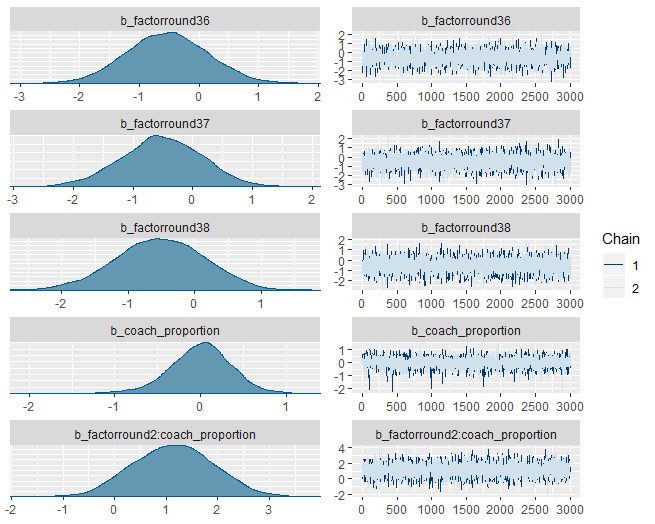


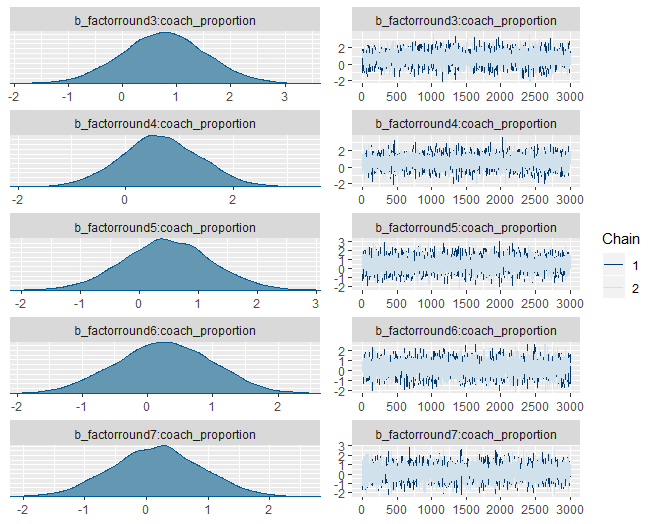


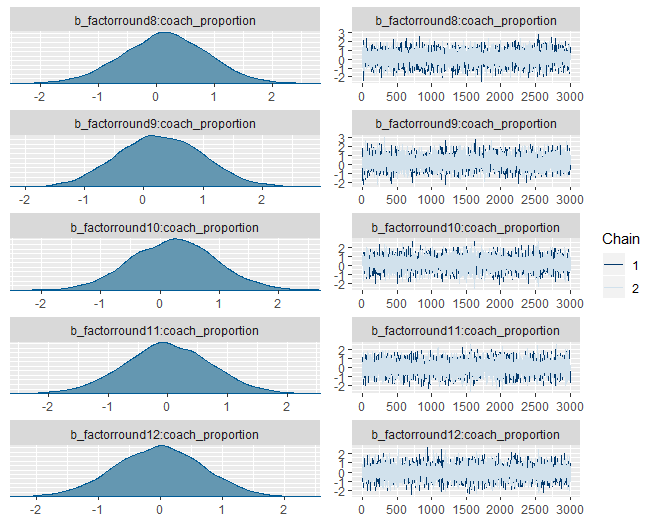


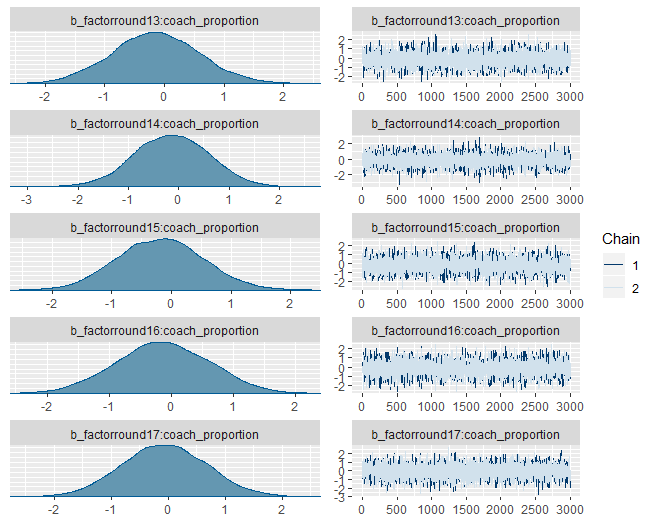


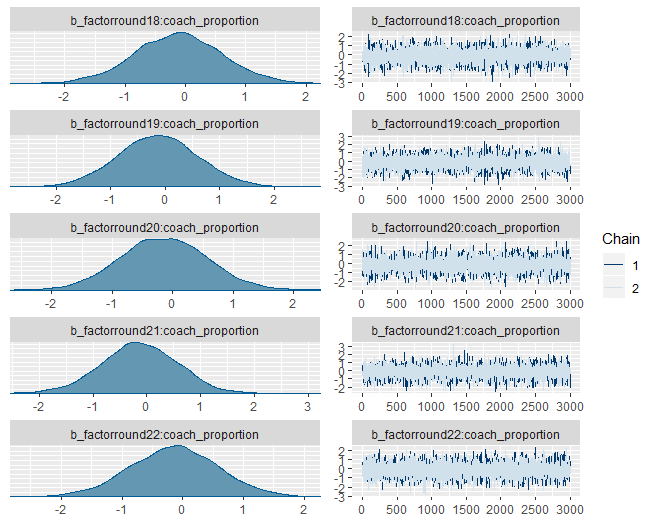


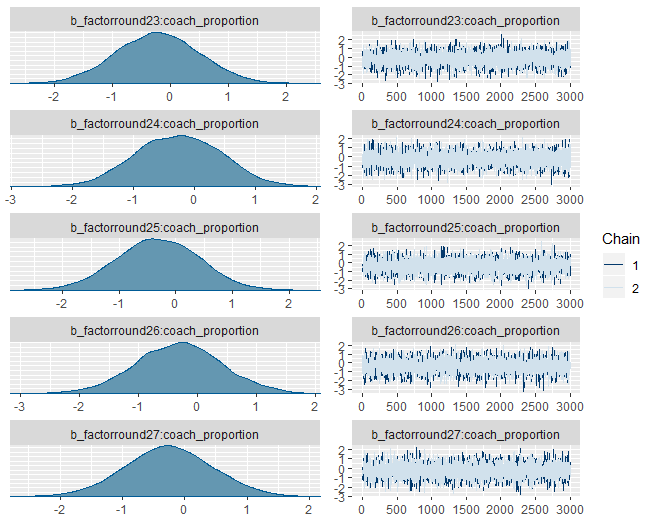


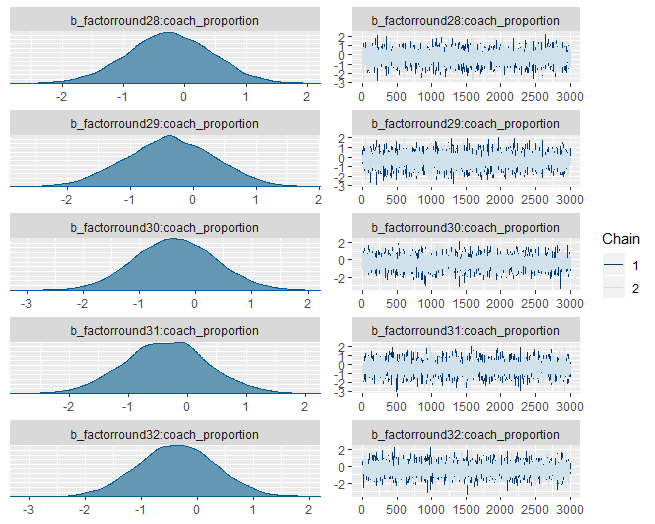


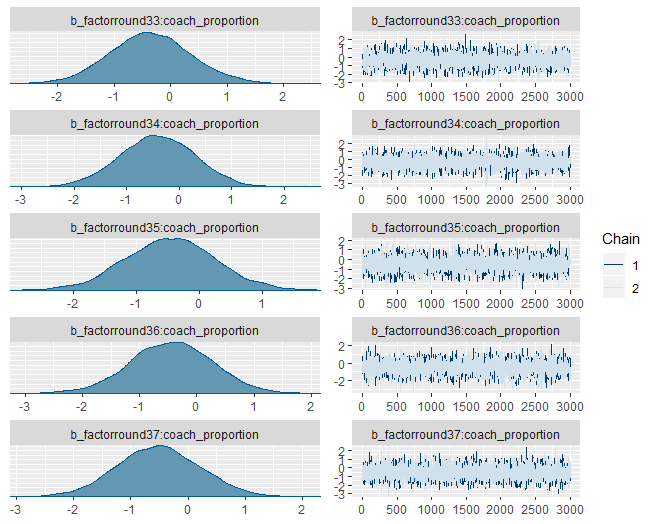


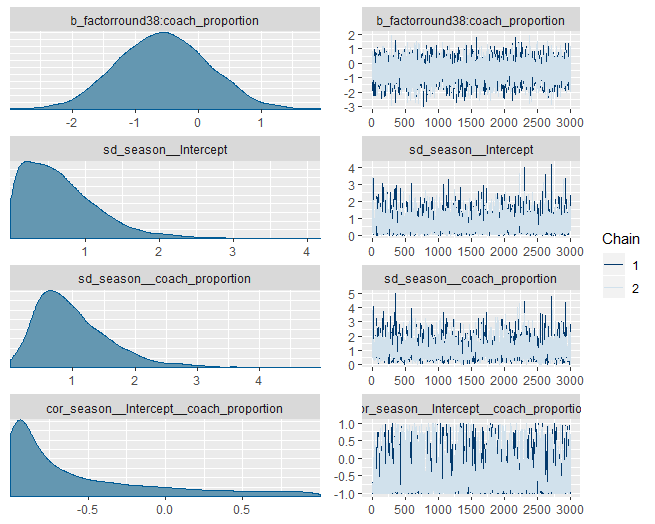


#posterior predictive checks

pp_check(m3,nsamples = 100)


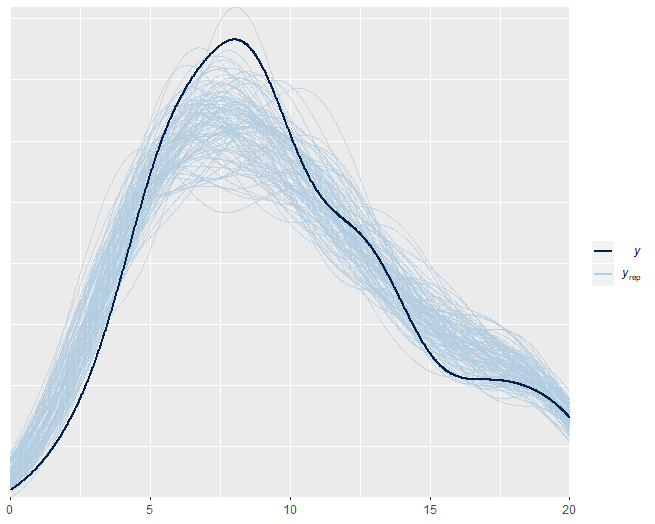

Supplement: Supplementary file 1 [file Table_1.DOCX]
